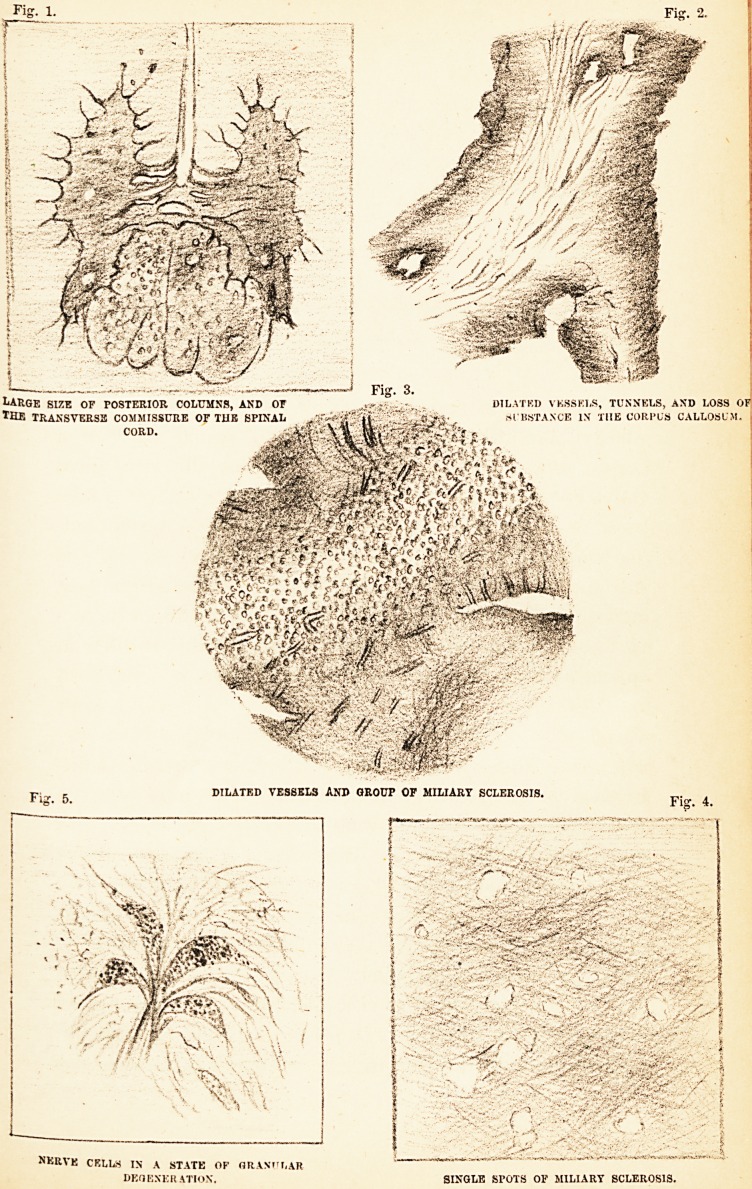# Notes on Some Morbid Appearances in the Brain and Spinal Cord of a Lioness

**Published:** 1885-12

**Authors:** W. B. Kesteven

**Affiliations:** Enfield, Middlesex


### NOTES ON SOME MORBID APPEARANCES IN THE BRAIN AND SPINAL CORD OF A LIONESS.

BY

W. B. KESTEVEN, M.D.,

*Enfield, Middlesex.*

---

A FEW months ago I received from Adelaide, South Australia, portions of the brain and spinal cord of a lioness that had died with paralysis, at the Zoological Gardens of that city. The portions of the brain were taken from the posterior convolutions of the hemispheres, including the descending limb of the corpus callosum. Of the spinal cord, there was only a length of about three inches, including the medulla oblongata. The general characters of these portions of the nervous centres did not differ in appearance from those of the normal cord and brain in man, horse, sheep, calf, cat, and monkey, beyond what would arise out of the relative difference in size of the animals. There were, however, a few microscopical differences in the cord of the lioness which may here be described. The most obvious of these were observed in the size of the posterior columns, particularly of the wedge-shaped columns of Goll, which parts were not only larger, but the medullary fibres of which they were composed were unusually large, and their sheaths and axis cylinders more distinct than usual. (Fig. I.) Besides these features, there were visible therein numerous points of miliary degeneration. The central commissure of the cord was broad, and exhibited strong bands of nerve fibres

Fig. 1.

LARGE SIZE OF POSTERIOR COLUMNS, AND OF THE TRANSVERSE COMMISSURE OF THE SPINAL CORD.

Fig. 2.

DILATED VESSELS, TUNNELS, AND LOSS OF SUBSTANCE IN THE CORPUS CALLOSUM.

Fig. 3.

DILATED VESSELS AND GROUP OF MILIARY SCLEROSIS.

Fig. 5.

NERVE CELLS IN A STATE OF GRANULAR DEGENERATION.

Fig. 4.

SINGLE SPOTS OF MILIARY SCLEROSIS.

passing off into the cornua. A similar arrangement may be seen in the spinal cord of the horse. (Fig. 1.) The anterior half of the central commissure was notably wider than the posterior segment, whilst the central canal was larger than in the human cord; its lumen circular and spacious. The multipolar cells in the cornua did not differ materially in size or form from those of the human cord. The aspect of their nuclei and processes presented no diversity as to number or size, except where they had undergone granular degeneration. (Fig. 5.)

The measurements of the cells in the anterior horn were as follow: Width, from $\frac{1}{200}$th to $\frac{1}{2166}$th inch; length, $\frac{1}{218}$th to $\frac{1}{500}$th inch.

The vessels, more especially in the corpus callosum, and convolutions, gave evidence of repeated distension during life, with *post-mortem* contraction, leaving what have been termed "perivascular spaces."

Tunnels and holes were also observable in the cerebral substance, from whence vessels had disappeared. (Figs. 2 and 3.) In some parts extensive loss of substance was visible.

To these indications of chronic disease were to be added groups of spots of miliary degeneration in the medullary substance of the brain (Fig. 3), as well as in the grey substance of the cord, together with fragments of myelin—the myelin sheath of the nerve fibres being very distinct, and the axis cylinders strongly marked. (Fig. 1.)

The appearances which are here referred to, (Fig. 4) as miliary "sclerosis," or degeneration, have been held by distinguished pathologists (prominently so by Dr. Savage, of Bethlem Royal Hospital) to be purely *post-mortem* changes, attributable to alcohol, or other hardening agents. I am not convinced of the correctness of this opinion, but it is only right to state that the brain and cord now under consideration had been preserved in spirits of wine. I may add, however, that a portion of cord of a calf, that had been in spirit about the same length of time, presented no trace of this lesion.

With much deference to the opinions of the eminent pathologists above alluded to, I would submit that, even if owing in any degree to the action of hardening agents, there must be a pre-existing morbid condition to account for its presence in some cases and its absence from others, under the employment of similar hardening agents. Further, I may say that bodies, in all respects resembling miliary sclerosis, if present, may be detected by staining media, in perfectly fresh substance of the brain and spinal cord.*

* See Dr. Batty Tuke, "On a handy Method of Examining Morbid Nervous Tissues Microscopically," *British Medical Journal*, Sept. 5th, 1874.

## Figures and Tables

**Fig. 1. Fig. 2. Fig. 3. Fig. 4 Fig. 5. f1:**